# Social license for the use of big data in the COVID-19 era

**DOI:** 10.1038/s41746-020-00342-y

**Published:** 2020-10-02

**Authors:** James A. Shaw, Nayha Sethi, Christine K. Cassel

**Affiliations:** 1 Joint Centre for Bioethics, University of Toronto, Toronto, ON Canada; 2 Institute for Health System Solutions and Virtual Care, Women’s College Hospital, University of Toronto, Toronto, ON Canada; 3 Centre for Biomedicine, Self and Society, Usher Institute, University of Edinburgh, Edinburgh, Scotland; 4 University of California San Francisco, San Francisco, CA USA

**Keywords:** Health policy, Medical ethics

## Abstract

Strategies to enable the reopening of businesses and schools in countries emerging from social-distancing measures revolve around knowledge of who has COVID-19 or is displaying recognized symptoms, the people with whom they have had physical contact, and which groups are most likely to experience adverse outcomes. Efforts to clarify these issues are drawing on the collection and use of large datasets about peoples’ movements and their health. In this Comment, we outline the importance of earning social license for public approval of big data initiatives, and specify principles of data law and data governance practices that can promote social license. We provide illustrative examples from the United States, Canada, and the United Kingdom.

Understanding the transmission, prevalence, and clinical characteristics of COVID-19 is essential for relaxing social distancing, shelter-in-place, and other policies carrying significant detrimental effects on national economies. Many countries are finding ways to expand large datasets that, with the support of advanced analytics and artificial intelligence, promise to answer crucial questions about the disease and its spread^1^. These initiatives raise challenges related to ethics, governance, and public attitudes. If these challenges are not addressed in ways that are transparent and broadly satisfying to the public, they risk public criticism, loss of public trust, and potentially premature shut down^2^.

We define the concept of social license and describe its relevance for health-related big data initiatives such as those proposed to mitigate the spread of COVID-19. We propose trust-enhancing governance practices that can increase trustworthiness in data gathering for public health purposes, drawing on experience in Canada, the United Kingdom and the United States.

## Social license matters for big data initiatives

Although every data initiative must give due regard to privacy and security requirements as prescribed within legislation, an equally important yet distinct set of concerns relates to social license of data uses. Here, we focus on the latter, arguing that it is particularly important given the reliance on public uptake of contact tracing apps and similar technologies to be effective as public health interventions.

Social license refers to the informal permissions granted to institutions such as governments or corporations by members of the public to carry out a particular set of activities^3,4^. Much of the literature on the topic of social license has arisen in the field of natural resources management, emphasizing issues that include but go beyond environmental stewardship^4^. In their seminal work on social license in the pulp and paper industry, Gunningham et al. defined social license as the “demands and expectations” placed on organizations by members of civil society which “may be tougher than those imposed by regulation”; these expectations thereby demand actions that go beyond existing legal rules to demonstrate concern for the interests of publics^5^. We use the plural term “publics” as opposed to the singular “public” to illustrate that stakeholder groups to which organizations must appeal are often diverse and varied in their assessments of whether a given organizational activity is acceptable^6^. Despite the potentially fragmented views of various publics, the concept of social license is considered in a holistic way (either an organization has it or does not). Social license is closely related to public trust, and where publics view a particular institution as trustworthy it is more likely to have social license to engage in activities such as the collection and use of personal data^7^.

The question of how the leaders of an organization might better understand whether they have social license for a particular set of activities has also been addressed in the literature. In a review of literature on social license, Moffat et al. highlighted disagreement in the research community about whether social license can be accurately measured^4^. Certain groups of researchers emphasize that because of the intangible nature of social license, accurate measurement will never truly be possible. Others propose conceptual models of the determinants of social license, and establish surveys that assess those determinants to indicate the presence or absence of social license in a given context. However, accurate measurement of social license remains a point of debate.

Literature on social license related to health care is sparse, despite conflicting evidence about the extent to which publics consider the individuals, organizations, and systems that constitute health care to be trustworthy^8,9^. However, a small body of literature exists related to social license and the use of personal health information for health-related research and policymaking;^3^ we provide select examples in the following section. In relation to initiatives seeking to build and leverage large datasets to better understand the nature and spread of COVID-19, certain governance strategies are more likely to have a positive effect on public trust and thereby promote the social license to enable their use. Decisions around data use must align with the social license granted to particular institutions to compile and use data for public health purposes during the pandemic^3^.

## Public trust in health data sharing

Many people are supportive of their health-related data being shared to support research and public health policy when certain conditions apply. A recent review of the literature summarized conditions that must be met from perspectives of various members of the public, including that health data are used for public benefit, in transparent ways, and by trusted institutions^10^. The recent experience of “Project Nightingale”, a partnership between Ascension Health System and Google to use advanced analytics to gain insights into patient data, is illustrative of what happens when transparency and trustworthiness are lacking. The apparent secrecy of the project motivated a powerful public backlash that ultimately led to a formal investigation by the US Department of Health and Human Services^2^. Projects in the United Kingdom have had a similar fate, such as the abandonment of the “care.data initiative” to collate data from primary care practices across the country as a result of resistance from health care providers and the public^3^.

The experiences documented in these projects may be contrasted with other health-related big data initiatives that have not been deemed as problematic by publics. For example, Mayo Clinic in Rochester, Minnesota struck a 10-year collaboration with Google to support their digital transformation of care; Wachter and Cassel suggested that the lack of controversy surrounding this particular initiative was due to its transparency^2^. The collaboration was made public before any data sharing occurred. Although no process exists to specify the extent to which the Mayo Clinic-Google collaboration enjoyed social license where the Ascension-Google collaboration did not, the public outcry arising from investigative journalism that revealed the latter to the public is an important consideration. In our view, the transparent and forthcoming nature of the announcement regarding the Mayo Clinic-Google collaboration allowed for less distrust and related backlash among the public.

Although conditions demonstrating transparency and trustworthiness can be met by certain health data analytics initiatives^2^, not all communities will agree on the nature of the conditions that must be met. One issue that requires special attention in any effort to collect and use personal data related to the COVID-19 pandemic is the informed mistrust of health systems by particular communities. For example, histories of structural racism and other exclusionary practices in health care in the United States have led to warranted suspicion and avoidance of mainstream health care by African Americans^11^. Other communities have also faced biases and systematic barriers to fair treatment in health care, including Indigenous Peoples, those with disabilities, people living with homelessness, and other marginalized groups. Many of these communities are the same ones who have been disproportionately affected by COVID-19, and controlling the spread of COVID-19 requires strategies that appeal to these communities. Earning social license to engage in activities such as digitally enabled contact tracing therefore requires special attention to information needs, opinions, and potential unintended consequences for communities who bear disproportionate risk of being harmed or perceiving risk of harm.

## Data governance for social license in the COVID-19 era

Policy strategies to enable the reopening of businesses and schools in countries emerging from orders of shelter-in-place and similar social-distancing measures revolve around knowledge of who has COVID-19, the people with whom they have had physical contact, and which groups are most likely to experience adverse outcomes. This information is essential to prevent additional spikes in the number of COVID-19 cases while enabling people to engage in a modified (i.e., socially distanced) version of everyday activities. Three strategies involving large health related datasets are central to these aims. First, using data about the proximity of known COVID-19 cases to other members of the public via mobile phones (digitally enabled contact tracing); second, forecasting specific areas more likely to experience outbreaks; and third, better understanding which proportions of populations are most likely to need high resource care. These data platforms can be used to aid in resource allocation such as ICU beds in a city during a surge.

The ways in which the data in each of these examples are acquired, used and governed vary considerably. For example, in some jurisdictions the use of digital contact tracing applications is mandatory (guaranteeing the generation of a related dataset), whereas in others it is voluntary (relying on express consent associated with the use of the application). Conversely, a majority of countries have infrastructure to collect data regarding basic characteristics of members of the public who have tested positive for COVID-19, enabling the generation of large population-wide datasets of known cases. Despite the variability in models of consent and data collection across these particular initiatives, we suggest that the links between data governance strategies and the attainment of social license are strong in all cases.

Although data governance strategies that exemplify trustworthiness are not alone sufficient to earn social license, we suggest that by demonstrating trustworthiness, good data governance can promote the attainment of social license. We propose that governance practices to promote trustworthiness and thereby promote the likelihood of attaining social license in these initiatives involve three interrelated yet distinct key features. In what follows, we outline principles of data privacy law and the data governance practices they recommend. The data privacy law principles we outline are distinct from social license as a concept, however abiding by these principles and the data governance practices they recommend can enhance the likelihood of earning social license in a given data initiative. We have selected these principles of focus for their commonality across many jurisdictions internationally and their links to the concept of social license outlined earlier.

First, the data used ought to be only those that are essential to achieve the specific public health goal of the initiative; this refers to the data privacy law principle of purpose limitation. For example, Article 5(1) of the General Data Protection Regulation demands that data processing is “adequate, relevant and limited to what is necessary in relation to the purposes for which they are processed^12^.” Purpose limitation is similar to but different from the related principle of data minimization, which refers to the act of limiting the type and quantity of data collected specifically to that which is necessary to perform the intended analysis. In the case of initiatives to compile and use data during the pandemic, clear statements of the uses to which data will be put can enhance the trustworthiness of the initiative involving the analysis of the data, thereby enhancing the likelihood of securing social license.

However, limiting the data used in the analyses outlined earlier (digital contact tracing, forecasting outbreaks, and allocating resources) also presents challenges. For example, excluding particular data elements from model development can result in a biased model that would have been more accurate and effective if trained on a broader dataset. Strategies to address these challenges, such as purposefully selecting and retaining those variables that might cause bias in the model if removed, will contribute to effective model development while maintaining the importance of good data governance practices that promote social license.

Second, we suggest that governance practices necessitate transparency. Specifically publics must be kept informed on the progress and plans of data use initiatives and ongoing decisions made. Especially during the continually evolving effort to control the spread of COVID-19 while reopening businesses and other sectors of the economy, establishing strategies to regularly inform publics on a large scale is essential. Regular updates about data sharing and uses, organizations involved, and the input received from public advisory bodies would bolster the effort to acquire and maintain social license for such initiatives. Acknowledging the challenges associated with the transparent provision of information to publics about conceptually difficult topics such as machine learning, we suggest that expert input into educational strategies to accomplish this goal would enhance the likelihood of attaining social license for digital contact tracing, forecasting spread of COVID-19, and informing the allocation of resources.

Finally, we suggest that sustained commitment to public involvement is crucial. This must include but go beyond strategies to educate the public, which are often viewed as paternalistic, one-directional and “top-down”. Although raising awareness of data uses is important for improving public understandings of data use, meaningful public engagement involves two-way, ongoing communication with publics in order to explore attitudes towards data uses and to reflect these concerns within governance frameworks adopted^13^.

Precedent exists around the world for the data governance practices we have described here. For example, the Public Benefit and Privacy Panel in Scotland includes public representatives in addition to other stakeholders who scrutinize applications to use National Health Services (NHS) Scotland health data. At the Institute of Clinical Evaluative Sciences in Ontario, Canada (a large health data repository), a Public Advisory Council provides input on a variety of data access and analysis initiatives. These groups are in place to ensure that data uses are appropriate from the perspective of the public interest, and to provide a mechanism for public involvement in data governance. Although there has not historically been great investment in large-scale transparency for big data initiatives^14^, it is clear that promoting social license during the pandemic will require it.

Drawing on insights from past successes and failures of data governance around the world, governments and their partners can ensure health data is used in ways that are legally sound and acceptable to the public. Doing so is essential to mobilizing large datasets in ways that contribute to the control of and response to the COVID-19 pandemic.

## Data Availability

Data sharing not applicable to this article as no datasets were generated or analyzed during the current study.

## Code availability

Code sharing not applicable to this article as no code was generated or used during the current study.
